# A cluster randomised trial of a school-based resilience intervention to decrease tobacco, alcohol and illicit drug use in secondary school students: study protocol

**DOI:** 10.1186/1471-2458-12-1009

**Published:** 2012-11-21

**Authors:** Rebecca K Hodder, Megan Freund, Jenny Bowman, Luke Wolfenden, Elizabeth Campbell, Paula Wye, Trevor Hazell, Karen Gillham, John Wiggers

**Affiliations:** 1 Hunter New England Population Health, Hunter New England Local Health District, Locked Bag 10, Wallsend, NSW 2287, Australia; 2 The University of Newcastle, Callaghan, NSW 2308, Australia; 3 Hunter Medical Research Institute, Locked Bag 1, Hunter Region Mc, NSW 2310, Australia; 4 Hunter Institute of Mental Health, Hunter New England Area Health Service, Locked Bag 10, Wallsend, NSW 2287, Australia

## Abstract

**Background:**

Whilst schools provide a potentially appropriate setting for preventing substance use among young people, systematic review evidence suggests that past interventions in this setting have demonstrated limited effectiveness in preventing tobacco, alcohol and other drug use. Interventions that adopt a mental wellbeing approach to prevent substance use offer considerable promise and resilience theory provides one method to impact on adolescent mental well-being. The aim of the proposed study is to examine the efficacy of a resilience intervention in decreasing the tobacco, alcohol and illicit drug use of adolescents.

**Methods:**

A cluster randomised controlled trial with schools as the unit of randomisation will be undertaken. Thirty two schools in disadvantaged areas will be allocated to either an intervention or a control group. A comprehensive resilience intervention will be implemented, inclusive of explicit program adoption strategies. Baseline surveys will be conducted with students in Grade 7 in both groups and again three years later when the student cohort is in Grade 10. The primary outcome measures will include self-reported tobacco, alcohol, marijuana and other illicit drug use. Comparisons will be made post-test between Grade 10 students in intervention and control schools to determine intervention effectiveness across all measures.

**Discussion:**

To the authors’ knowledge this is the first randomised controlled trial to evaluate the effectiveness of a comprehensive school-based resilience intervention, inclusive of explicit adoption strategies, in decreasing tobacco, alcohol and illicit drug use of adolescents attending disadvantaged secondary schools.

**Trial registration:**

ACTRN12611000606987

## Background

Globally, a significant proportion of people, including those in Australia, are at risk of harm from smoking, alcohol misuse or illicit drug use [1,2]. Of all age groups, young people report the greatest prevalence of such substance use [3]. The younger the age of initiation of substance use, the greater the likelihood of ongoing use, dependence and harm in later life [4]. As such, primary prevention efforts focusing on preventing initiation of substance use by young people have been recommended [5].

Schools provide an appropriate setting for improving the immediate and long term health of young people as adolescents [6-8], however there is limited evidence that school-based programs are effective [5,9,10]. A recent review of school-based programs targeting alcohol however has reported that interventions that aimed to develop the psychological or social skills of young people had the most promise [5,9]. The findings of these reviews are consistent with a World Health Organisation review that concluded that programs that promote young people’s mental well-being were most likely to be effective [11]. The same World Health Organisation review suggests that school-based interventions that address the school curriculum, school environment and community were the most likely to achieve a beneficial outcome, a method known as the Health Promoting Schools approach [11].

Resilience theory, which has arisen from the study of risk factors for, and their impact on, positive youth development represents one approach to improving adolescent mental or psychosocial well-being [12-18]. Whilst there is variation in the definition of resilience, it is generally agreed that both individual (internal) as well as environmental (external) characteristics contribute to individual resilience and are critical for positive youth development and the avoidance of risk behaviours [19-21]. An inverse association has been found to exist between adolescent resilience characteristics and substance use [22-24].

Although a number of school-based studies have reported targeting some aspect of adolescent resilience as a basis for intervention, none have applied the approach in a comprehensive manner nor have they demonstrated consistent effect [25-30]. In a number of such studies, the researchers have concluded that inadequate intervention dose and fidelity may have contributed to the limited outcomes [29]. A number of barriers to intervention adoption have been cited including: a lack of financial resources for planning, training, and teacher release; inadequate levels of professional development; inadequate program resources; failure to adopt a ‘whole of school’ approach to implementation and monitoring; and inadequate support by school executives [31].

A pilot study of a comprehensive intervention addressing both internal and external adolescent resilience factors in a convenience sample of three socio-economically disadvantaged secondary schools has recently been reported. The intervention was delivered using the Health Promoting Schools approach [32], and included explicit strategies to enhance intervention adoption such as adoption support staff, resource provision and staff training. The evaluation suggested significant increases across all three schools in internal and external resilience scores, and significant decreases across all three schools in prevalence of student smoking, alcohol consumption and marijuana use [32]. Such positive outcomes were demonstrated for all grades and genders, and exceeded declining temporal trends in the broader population [32].

Whilst the findings of the pilot study were positive, a more rigorous study design is required to confirm the potential of such a comprehensive resilience enhancing approach. A cluster randomized controlled trial is planned to examine the efficacy of a comprehensive resilience intervention, inclusive of intervention adoption strategies, in decreasing the tobacco, alcohol and illicit drug use of adolescents attending secondary schools in a socio-economically disadvantaged region.

## Methods

### Study design

**Figure 1:**
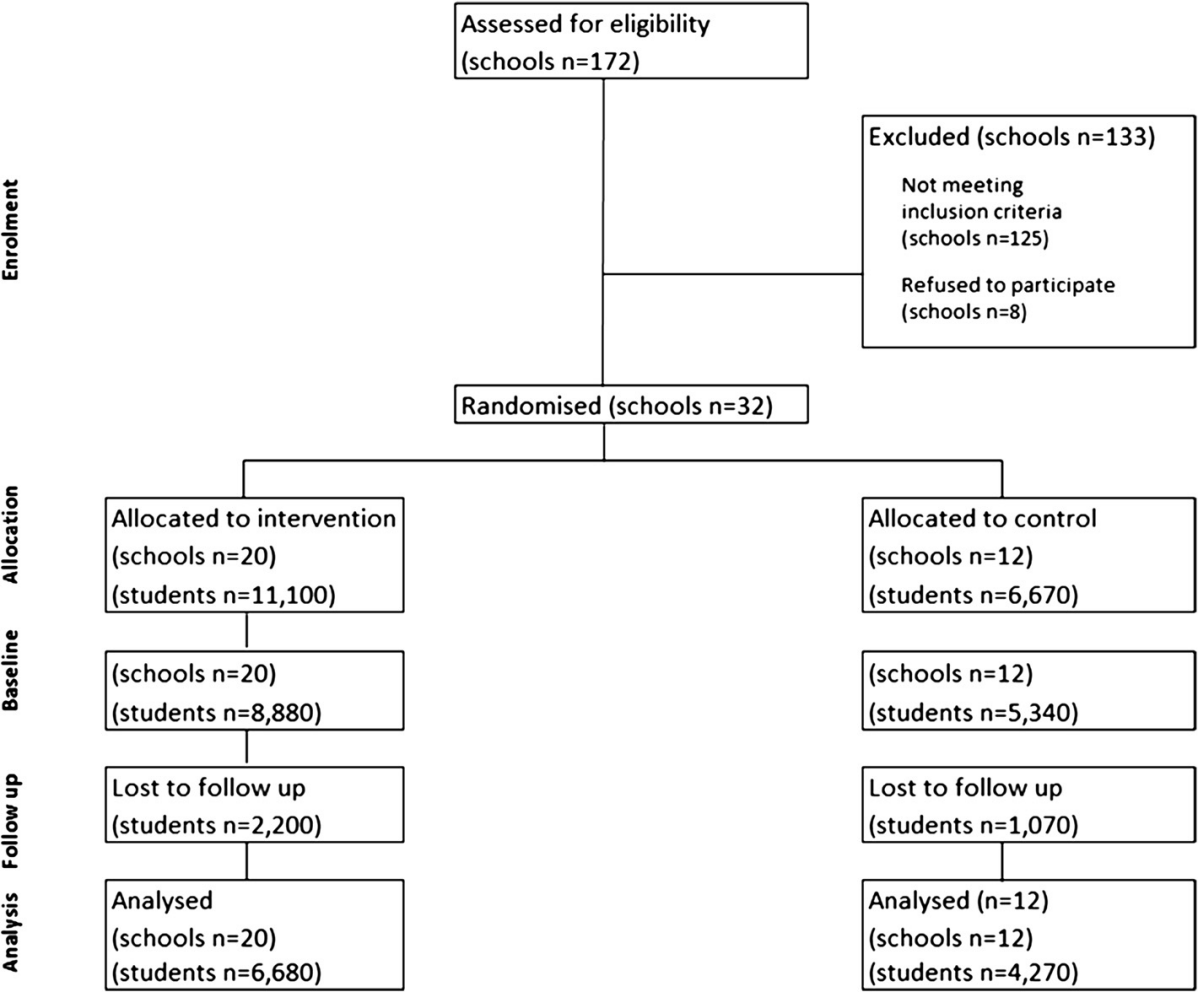
Estimated CONSORT flow diagram of the school progress through the phrases of the trial.

A cluster randomised control trial design (Figure 1) will be conducted, with schools as the unit of randomisation. Thirty two schools will be randomly selected to participate in the study, with 20 schools randomly allocated to the intervention group, and 12 to the control group. A resilience intervention will be implemented in schools allocated to the intervention group. Schools allocated to the control group will not receive any intervention during the study period. To assess the efficacy of the intervention, baseline web-based surveys will be conducted with Grade 7 students. Follow up data will be collected using the same method three years later when the original cohort of students are in Grade 10.

The trial has been approved by the Hunter New England Health Human Research Ethics Committee (Ref no. 09/11/18/4.01), The University of Newcastle Human Research Ethics Committee (Ref no. H-2010-0029), the Aboriginal Health and Medical Research Council (Ref no. 776/11), the New South Wales Department of Education and Training State Education Research Approval Process (Ref no. 2008118), and the relevant Catholic Schools Offices. In addition, the trial is registered with the Australia and New Zealand Clinical Trials Register (Ref no. ACTRN12611000606987).

### Participants

#### School sample

The study will be conducted in 32 government and Catholic secondary schools within the Hunter New England Local Health District of New South Wales, Australia. Approximately 135,000 adolescents reside in the Hunter New England Local Health District which encompasses metropolitan, regional, rural and remote locations across an area of 130,000 square km [33].

Schools will be selected from a list of all government and Catholic secondary schools in the study area obtained from the Department of Education and Training and from relevant regional Catholic School Offices. Schools will be eligible if they are located in a disadvantaged Local Government Area (as defined by the SEIFA Index of Relative Socio-Economic Advantage/Disadvantage by Local Government Area) [34], have more than 400 total student enrolments, and have enrolments in Grades 7 to 10. In addition, due to the likelihood of differential effects, schools which are entirely special needs or selective, central schools, boarding schools or are not co-educational schools are ineligible for inclusion in the trial.

#### School recruitment

Before being invited to participate in the trial, briefing meetings will be held to inform all school principals of the study. An eligibility interview will subsequently be conducted with each school principal to identify current implementation of resilience strategies. Schools considered to be implementing a comprehensive resilience intervention within each domain of the Health Promoting Schools framework across all grades will be ineligible and removed from the list of eligible schools.

The order in which eligible schools will be invited to participate will be determined using a random number function (in Microsoft Excel) by an independent statistician. The principals of the first 32 randomly selected eligible secondary schools will be sent a letter informing them about the study and requesting written consent for their school to participate. Within one week from the initial information letters being sent, research staff will contact non-responding principals to answer any questions they may have and to prompt their reply. Principals that do not reply within a further week will receive additional prompts from research staff. If a school declines to consent the next school on the list will be invited, following the same procedure above.

#### Random allocation of schools

Once 32 schools have been recruited to the study, the sample will be stratified by engagement in a national government initiative directed at schools in disadvantaged areas [35], and school size (medium sized school 400–800; large sized school >800). Schools will then be randomly allocated in a 20:12 block design ratio to either the intervention or the control group. Neither schools, parents of students, nor students attending selected schools will be blinded to the school allocation.

#### Student sample

All students in Grade 7 (first year at high school and aged 12 to 13 years) attending consenting schools will be eligible to participate in the study (approximately 3,600 students at baseline).

#### Student recruitment

Active parental consent will be required for child participation in the evaluation component of the study. A number of strategies will be employed to maximize parental consent for child participation based on a recent review of school-based recruitment methods [36]. Schools will be provided with information to disseminate via existing school communication channels, including school newsletters, assemblies, staff meetings, and school community and parent meetings. Parents of children will be mailed study information packs that include a short cover letter from the school principal on school letterhead, a detailed study information letter for parents, a simplified study information letter for students, a parental consent form for child participation in the study and a reply paid envelope. The information letter will also provide the details for a free call message service on which parents can leave their details if they do not want to be prompted for consent. Parents will be asked to return the consent form by either using the reply paid envelope or by providing it to their child’s school.

Two weeks after distribution of the information packs, non-responding parents will be telephoned by school affiliated staff to prompt return of the child consent form. During the phone call, parents will be asked if they consent to their child participating in the survey. Parents who provide verbal consent during this phone call will be mailed a replacement information sheet and consent form (with reply paid envelope) so that written consent can be obtained.

### Resilience intervention

#### Intervention content

A multi-strategy intervention, based on a range of programs and strategies that address both internal and external adolescent resilience factors will be implemented for the whole school. Schools will be asked to implement a range of strategies in each of the Health Promoting Schools domains (curriculum, teaching and learning; ethos and environment; and partnerships and services) to enhance student resilience [37]. The strategies schools select will be informed by a comprehensive and structured planning process and will be selected from a list of evidence based programs (eg. MindMatters program). In this manner, the strategies implemented by each school may differ. However, schools will be asked to implement strategies to an intervention standard (see Table 1).

**Table 1:** Resilience intervention standards


**Curriculum, teaching and learning**	
·	100% of students in Grade 7-10 receive a minimum of 12 age-appropriate resilience lessons across subjects (e.g. implementation of MindMatters curriculum resources) [38]
·	100% of students in Grade 7-10 receive an additional 9 hours of non-curriculum based resilience programs (e.g. implementation of the ‘Resourceful Adolescent Program [34]
**Ethos and environment**	
·	Rewards and recognition program implemented across the whole school
·	Peer support/peer mentoring programs implemented across the whole school
·	Anti-bullying programs implemented across the whole school
·	Cultural awareness program implemented across the whole school
·	Teacher offered training to implement effective pedagogy within learning environments (e.g. MindMatters Teaching and Learning for Engagement) [38]
**Partnerships and services**	
·	Promotion and engagement of local community organisations/groups/clubs in the school (e.g. charity organisations, church or sporting groups)
·	Promotion and engagement of health and community services in the school (e.g. Youth and Child and Adolescent Mental Health Services)
·	School implement strategies to increase parental involvement in school (e.g. school events, effective parent communication strategies)
·	School promotes strategies to address students resilience at home (e.g. newletters regarding enhancing student resilience)

### Intervention adoption strategies

To increase the extent of intervention adoption and fidelity across schools, a number of strategies will be implemented that have been previously reported to facilitate adoption of school-based interventions, to change the service delivery practices of human service organizations, and to build capacity of organisations [17,31,39-44].

#### School intervention officers

A part-time school intervention officer will be located in each school to support data collection, and program planning, implementation and monitoring. Their role will be to support schools to implement the resilience intervention. School intervention officers will not implement intervention strategies directly with students. Such officers will be employed for three years and allocated to intervention schools at a ratio of one officer per four schools. Prior to intervention implementation, school intervention officers will undertake a two week intervention training program.

A school project coordinator will be employed full time for two years to support the implementation of the intervention at a regional level, provide support to the school intervention officers, and liaise with schools and the two education sectors.

#### Fiscal resources

In each year of the intervention, schools will be provided with an annual allocation of AUS$2,000 seed funding to facilitate training, professional development and teacher release time for intervention implementation.

#### Leadership

A school core team will be established, or an existing team will be enhanced, at each school to lead the implementation of the project. The core team will include the allocated school intervention officer, school staff and involvement of at least one member of the school executive.

#### Structured planning process

A structured planning process will be undertaken to design and implement the intervention in each school. A needs assessment will be undertaken immediately prior to intervention implementation to inform the development of strategies targeting student resilience. This assessment will include a resilience factor and substance use survey of all students in Grades 7 to 10 and a school environment survey of school policies, practices and curriculum that may impact on student resilience.

Planning workshops will then be held at each school to prioritize student resilience needs and identify feasible strategies to address them. Following this, each school will develop an individual intervention plan which will be endorsed by the school executive and integrated into existing student welfare governance processes and school planning. Schools will be provided with an implementation guide that describes in detail the steps required to implement this planning process, inclusive of available resilience programs, tools and templates.

#### Training

Key school staff will be provided with training in the planning, delivery and monitoring of the resilience intervention strategies (including resilience professional development). All school staff will be offered training in effective pedagogy for enhancing student resilience [45], and mental health literacy [46].

#### Monitoring and feedback

The school intervention officer will be responsible for monitoring and maintaining project records. School-specific feedback regarding intervention progress and data collected via student surveys will be provided to each. Intervention progress will also be provided to senior managers from the New South Wales Department of Education and Training and the relevant regional Catholic School Offices.

### Control group

Schools allocated to the control group will receive a report of their student survey data in the first and fourth year of the study. In addition, control schools will be given all printed intervention resources at the conclusion of the trial.

### Data collection procedures

#### Student outcomes

Baseline data will be collected via a student survey completed by all consenting students in Grade 7 in both intervention and control schools in the first year of the study. Follow up data will be collected from the same students in the fourth year of the study when the student cohort is in Grade 10 in the same season as baseline.

The student survey will take approximately 25 minutes to complete. Data collection staff will be recruited and trained to support students to complete the online survey during class time.

#### School outcomes

At baseline and follow up, a school environment survey will be undertaken with key school staff including principals and head teachers of both intervention and control schools to identify existing school policies and practices that target student resilience.

### Measures

#### Student demographics

The student survey will include the following demographic items: age, Grade, gender, ethnicity and Aboriginal and Torres Strait Islander status, languages spoken at home, and postcode.

#### Primary outcome measures – substance use

The outcome measures will be student-reported smoking, alcohol use and illicit drug use.

For both recent tobacco and alcohol use, retrospective diaries will be used to measure consumption in the past 7 days [25,47,48]. Recent use will be defined as having smoked any cigarettes or consumed any alcoholic drinks in the last week. These items are used regularly in Australian state-wide surveys of secondary school student health behaviours [49].

For risky alcohol consumption, the Australian guidelines recommend that there is no safe drinking level for adolescents [50]. As such, a measure of risky drinking based upon the Australian recommendations for adult risk for injury (no more than 4 standard drinks on one occasion) will be used [50].

For illicit drug use, data regarding marijuana and any other illicit drug use in the past month will be collected [49]. The items are used regularly in state-wide surveys of secondary school student health behaviours [49].

Data will also be collected regarding known moderators of tobacco and alcohol use, including sibling use, parent/carer use, access, receipt of pocket money or income from paid employment, and belief health will be damaged by tobacco and alcohol use [51,52].

In a selection of schools, a 10% random selection of students whose parents have provided consent will be asked to provide a saliva sample at the completion of the student survey in the fourth year of the study. The saliva sample will be used to test for the presence of cotinine (a metabolite of nicotine) to validate the accuracy of their self-reported tobacco use [53]. All students attending these schools will be informed that they may be selected to provide a saliva sample to confirm their smoking status (a variation of the bogus pipeline method) [54]. Although the accuracy of cotinine assessment may be influenced by less frequent adolescent smoking, it is the most suitable measure given its low level of personal intrusion, portability and cost [55].

#### Secondary outcome measures - resilience

Items from the California Healthy Kids Survey will be used to measure student internal and external resilience. The survey addresses six internal resilience factor subscales and eight external resilience factor subscales which have demonstrated adequate reliability [31]. The internal resilience subscales include Likert scale items addressing: cooperation and communication (2 items); self-efficacy (4 items); empathy (3 items); problem solving (3 items); self-awareness (3 items); goals and aspirations (3 items); school support (6 items); school meaningful participation (3 items); community support (6 items); community meaningful participation (3 items); home support (6 items); home meaningful participation (3 items); peer caring relationships (3 items); pro-social peers (3 items).

#### School environment survey

The school environment survey will be developed by the research team and will aim to measure the programs, curriculum, policies, practices, and partnerships that schools are currently implementing that target student resilience.

### Sample size

Based on past research [7,56], and the pilot study results, an estimated 80% of students will consent to participate. Based on this, and an estimated loss of students to follow-up from Grade 7 to Grade 10 of 25% a cohort sample of 1,360 Grade 7 students and 1,020 Grade 10 students in the control condition, and 2,270 Grade 7 students and 1,700 Grade 10 students in the intervention condition, will be the subject of analysis. Assuming 80% power, a 5% significance level, an intra-cluster correlation of 0.01, and a Grade 10 control group smoking prevalence of 14% the study will be able to detect a 4.8% lower prevalence of smoking for the intervention group. For recent and risky alcohol consumption, assuming a Grade 10 control group prevalence of 36.2%, a 7.0% lower prevalence of consumption can be detected. For use of marijuana, assuming a Grade 10 control group prevalence of 25%, a 6.2% lower prevalence of use respectively can be detected for marijuana. Finally, for other illicit drugs, assuming a Grade 10 control group prevalence of 9.3%, a 3.9% lower prevalence of use can be detected for the intervention group.

### Statistical analysis

All analyses will be undertaken using SAS Software Version 9.2 [57].

#### Demographic characteristics

Intervention and control school parental consent rates will be examined for non-response bias using Chi square analysis. Intervention and control school student demographic characteristics will be compared at baseline and follow up also using Chi square analysis.

#### Primary outcomes – substance use

Comparisons in prevalence of substance use will be made between Grade 10 students in intervention and control schools at follow up to determine intervention effectiveness for each outcome measure. Generalized linear models (generalized estimating equation (GEE approach)) will be used [58]. The GEE will cater for dichotomous outcome data, school clustering, levels of strategy implementation, and student or school characteristics that are potential confounding variables. Usual assumptions of linear regression will be tested (including linearity, independence, constant variance and normality), with variations from normality adjusted for using the robust variance estimator. For all outcome analyses, school will be included in the model as the clustering unit.

#### Secondary outcomes - resilience

Assuming resilience scores are not normally distributed (as with the data from the pilot study) comparison of median scores will be made post-test between Grade 10 students in intervention and control schools to determine intervention effectiveness. Linear regression models (GEE) will test differences in overall sub-scale levels of resilience factors (predictor variable being treatment group).

#### School environment survey outcomes

Existing resilience intervention implementation collected via the school environment surveys in both intervention and control groups will be compared at both baseline and follow up using descriptive statistics.

#### Implementation cost

Costs will be calculated with the support of a health economist from the perspective of routine school delivery (development and research costs will be excluded). The proposed costing approach has previously been successfully undertaken and reported by the study team [59,60].

## Discussion

To the authors’ knowledge this is the first randomised controlled trial to evaluate the effectiveness of a comprehensive school-based resilience intervention, inclusive of explicit adoption strategies, in decreasing the self-reported tobacco, alcohol and illicit drug use of adolescents attending disadvantaged secondary schools. The intervention has been found to be feasible and acceptable when piloted in three secondary schools.

The current study offers the opportunity to provide evidence that a resilience approach is effective in addressing adolescent substance use in line with the recent reviews that suggest this. If the results indicate the resilience intervention is effective in decreasing the prevalence of adolescent substance use, a new and strengthened intervention model will be available to aid policy makers and educationalists in implementing interventions targeting adolescent substance use, inclusive of program adoption strategies.

## Competing interests

There are no competing interests for any of the authors of this manuscript.

## Authors’ contributions

RKH drafted the manuscript; and participated in the conception, design and coordination of the study. MF, JB, EC, KG and JW helped draft the manuscript and participated in the conception, design and coordination of the study. LW, PW and TH helped draft the manuscript and participated in the conception and design of the study. All authors read and revised the manuscript critically for intellectual content, and approved the final manuscript.

## Pre-publication history

The pre-publication history for this paper can be accessed here:

http://www.biomedcentral.com/1471-2458/12/1009/prepub

## References

[B1] Lopez AD Global burden of disease and risk factors 2006 Washington D.C: Oxford University Press

[B2] Australian Institute of Health and Welfare 2007 National Drug Strategy Household Survey: First Results 2008 Canberra: AIHW

[B3] Australian Institute of Health and Welfare Young Australians: their health and wellbeing 2007 2007 Canberra: AIHW

[B4] Gilman SE Abrams DB Buka SL Socioeconomic status over the life course and stages of cigarette use: initiation, regular use, and cessation J Epidemiol Community Health 2003 57 802 808 10.1136/jech.57.10.802 14573586 PMC1732304

[B5] Faggiano F Vigna-Taglianti FD Versino E Zambon A Borraccino A Lemma P School-based prevention for illicit drugs' use Cochrane Database Syst Rev 2005 2 CD003020 15846647 10.1002/14651858.CD003020.pub2

[B6] Wyn J Cahill H Holdsworth R Rowling L Carson S MindMatters, a whole-school approach promoting mental health and wellbeing Aust N Z J Psychiatry 2000 34 594 601 10.1080/j.1440-1614.2000.00748.x 10954390

[B7] Patton GC Bond L Carlin JB Thomas L Butler H Glover S Catalano R Bowes G Promoting social inclusion in schools: a group-randomized trial of effects on student health risk behavior and well-being Am J Public Health 2006 96 1582 1587 10.2105/AJPH.2004.047399 16873760 PMC1551970

[B8] New South Wales Department of Education and Training New South wales Department of Education and Training internet Site 2008 Sydney: New South Wales Department of Education and Training

[B9] Foxcroft DR Tsertsvadze A Universal school-based prevention programs for alcohol misuse in young people Cochrane Database Syst Rev 2011 5 CD009113 21563171 10.1002/14651858.CD009113

[B10] Thomas R Perera R School-based programmes for preventing smoking Cochrane Database Syst Rev 2006 3 CD001293 16855966 10.1002/14651858.CD001293.pub2

[B11] Stewart-Brown S What is the evidence on school health promotion in improving health or preventing disease and, specifically, what is the effectiveness of the health promoting schools approach? 2006 Copenhagen: WHO Regional Office for Europe (Health Evidence Network report)

[B12] Catalano RF Berglund ML Ryan JAM Lonczak HS Hawkins JD Positive youth development in the united states: research findings on evaluations of positive youth development programs Ann Am Acad Pol Soc Sci 2004 591 124

[B13] Fergus S Zimmerman MA Adolescent resilience: a framework for understanding healthy development in the face of risk Annu Rev Public Health 2005 26 399 419 10.1146/annurev.publhealth.26.021304.144357 15760295

[B14] Greenberg MT Weissberg RP O'Brien MU Zins JE Fredericks L Resnik H Elias MJ Enhancing school-based prevention and youth development through coordinated social, emotional, and academic learning Am Psychol 2003 58 6–7 466 474-Jul 12971193 10.1037/0003-066x.58.6-7.466

[B15] Greenberg MT Promoting resilience in children and youth: preventive interventions and their interface with neuroscience Ann N Y Acad Sci 2006 1094 139 150 10.1196/annals.1376.013 17347347

[B16] Harvey J Delfabbro PH Psychological resilience in disadvantaged youth: A critical overview Australian Psychologist 2004 39 3 13 10.1080/00050060410001660281

[B17] Luthar SS Cicchetti D Becker B The construct of resilience: a critical evaluation and guidelines for future work Child Dev 2000 71 543 562 10.1111/1467-8624.00164 10953923 PMC1885202

[B18] Masten AS Ordinary magic. Resilience processes in development Am Psychol 2001 56 227 238 11315249 10.1037//0003-066x.56.3.227

[B19] Benard B Fostering resiliency in urban schools 1996 Virginia: Association for Supervision and Curriculum Development

[B20] Bernat DH Resnick MD Healthy youth development: science and strategies J Public Health Manag Pract 2006 Suppl S10 S16 17035894 10.1097/00124784-200611001-00004

[B21] Toumbourou JW Drug prevention strategies: A developmental settings approach prevention research evaluation report number 2 2002 Melbourne: Australian Drug Foundation

[B22] Bond L Butler H Thomas L Carlin J Glover S Bowes G Patton G Social and school connectedness in early secondary school as predictors of late teenage substance use, mental health, and academic outcomes J Adolesc Health 2007 40 357 e9 e357 e18 17367730 10.1016/j.jadohealth.2006.10.013

[B23] Resnick MD Bearman PS Blum RW Bauman KE Harris KM Jones J Tabor J Beuhring T Sieving RE Shew M Ireland M Bearinger LH Udry JR Protecting adolescents from harm. Findings from the national longitudinal study on adolescent health JAMA 1997 278 823 832 10.1001/jama.1997.03550100049038 9293990

[B24] Wiefferink CH Peters L Hoekstra F Dam GT Buijs GJ Paulussen TG Clustering of health-related behaviors and their determinants: possible consequences for school health interventions Prev Sci 2006 7 127 149 10.1007/s11121-005-0021-2 16596470

[B25] Bond L Thomas L Coffey C Glover S Butler H Carlin JB Patton G Long-term impact of the gatehouse project on cannabis use of 16-year-olds in Australia J Sch Health 2004 74 23 29 10.1111/j.1746-1561.2004.tb06597.x 15022372

[B26] Foxcroft DR Ireland D Lister-Sharp DJ Lowe G Breen R Primary prevention for alcohol misuse in young people Cochrane Database Syst Rev 2002 3 CD003024 12137668 10.1002/14651858.CD003024

[B27] Gorman DM Conde E Huber JC Jr The creation of evidence in 'evidence-based' drug prevention: a critique of the strengthening families program plus life skills training evaluation Drug Alcohol Rev 2007 26 585 593 10.1080/09595230701613544 17943519

[B28] Perry CL Williams CL Veblen-Mortenson S Toomey TL Komro KA Anstine PS McGovern PG Finnegan JR Forster JL Wagenaar AC Wolfson M Project Northland: outcomes of a community wide alcohol use prevention program during early adolescence Am J Public Health 1996 86 956 965 10.2105/AJPH.86.7.956 8669519 PMC1380436

[B29] Piper DL Moberg DP King MJ The healthy for life project: behavioral outcomes J Prim Prev 2000 21 47 73 10.1023/A:1007005430924

[B30] Spoth RL Redmond C Trudeau L Shin C Longitudinal substance initiation outcomes for a universal preventive intervention combining family and school programs Psychol Addict Behav 2002 16 129 134 12079251

[B31] MindMatters Evaluation Consortium Report of the MindMatters (national mental health in schools project) evaluation project, vols. 1–4 2000 Newcastle: Hunter Institute of Mental Health

[B32] Hodder R Daly J Freund M Bowman J Hazell J Wiggers J A school-based resilience intervention to decrease tobacco, alcohol and marijuana use in high school students 2011 United Kingdom: BioMed Central 10.1186/1471-2458-11-722 PMC3203076 21942951

[B33] New England Local Health District: Health in Hunter New England HealtheResource 2010 Newcastle: Hunter New England Population Health;

[B34] Trewin D Information paper census of population and housing socio-economic indexes for areas: Australia 2001 2003 Canberra: Australian Bureau of Statistics: Commonwealth of Australia

[B35] NSW Department of Education and Communities Low socio-economic status school communities national partnership 2011 Sydney: New South Wales Department of Education and Training;

[B36] Wolfenden L Kypri K Freund M Hodder R Obtaining active parental consent for school-based research: a guide for researchers Aust N Z J Public Health 2009 33 270 275 10.1111/j.1753-6405.2009.00387.x 19630848

[B37] World Health Organisation Planning meeting in health promoting schools project: background, development and strategy outline of the health promoting schools project 1991 WHO, Copenhagen

[B38] MindMatters Evaluation Consortium MindMatters: A mental health promotion resource for secondary schools 2000 Commonwealth of Australia, Canberra

[B39] Bond L Glover S Godfrey C Butler H Patton GC Building capacity for system-level change in schools: lessons from the gatehouse project Health Educ Behav 2001 28 368 383 10.1177/109019810102800310 11380056

[B40] Gottfredson DC Gottfredson GD Quality of school-based prevention programs: results from a national survey Journal of Research in Crime and Delinquency 2002 39 3 36 10.1177/002242780203900101

[B41] Hazell T MindMatters: evaluation of the professional development program and school-level implementation 2006 Hunter Institute of Mental Health, Newcastle

[B42] Lezotte LW Skaife RD Holstead MD Effective schools: only you can make a difference 2002 All Star Publishing, San Francisco

[B43] Wagner EF Tubman JG Gil AG Implementing school-based substance abuse interventions: methodological dilemmas and recommended solutions Addiction 2004 99 Suppl-19 10.1111/j.1360-0443.2004.00858.x 15488109

[B44] Wilson KD Kurz RS Bridging implementation and institutionalization within organizations: proposed employment of continuous quality improvement to further dissemination J Public Health Manag Pract 2008 14 109 116 18287915 10.1097/01.PHH.0000311887.06252.5f

[B45] MindMatters Whole school matters - draft manuscript 2010 Canberra: Commonwealth of Australia

[B46] Orygen Youth Health Research Centre Mental health first Aid 2011 Orygen Youth Health: Melbourne

[B47] Kypri K Gallagher SJ Incentives to increase participation in an Internet survey of alcohol use: a controlled experiment Alcohol Alcohol 2003 38 437 441 10.1093/alcalc/agg107 12915520

[B48] Wang YC Lee CM Lew-Ting CY Hsiao CK Chen DR Chen WJ Survey of substance use among high school students in Taipei: web-based questionnaire versus paper-and-pencil questionnaire J Adolesc Health 2005 37 289 295 10.1016/j.jadohealth.2005.03.017 16182139

[B49] Centre for Epidemiology and Research New South Wales School Students Health Behaviours Survey: 2008 report 2009 Sydney: NSW Department of Health

[B50] National Health and Medical Research Council Australian guidelines to reduce health risk from drinking alcohol 2009 Canberra: Australian Government

[B51] Dielman TE Butchart AT Shope JT Miller M Environmental correlates of adolescent substance use and misuse: implications for prevention programs Int J Addict 1990 25 855 880 2131323 10.3109/10826089109071027

[B52] Tyas SL Pederson LL Psychosocial factors related to adolescent smoking: a critical review of the literature Tob Control 1998 7 409 420 10.1136/tc.7.4.409 10093176 PMC1751465

[B53] Montalto NJ Wells WO Validation of self-reported smoking status using saliva cotinine: a rapid semiquantitative dipstick method Cancer Epidemiol Biomarkers Prev 2007 16 1858 1862 10.1158/1055-9965.EPI-07-0189 17855706

[B54] Murray DM Perry CL The measurement of substance use among adolescents: when is the 'bogus pipeline' method needed? Addict Behav 1987 12 225 233 10.1016/0306-4603(87)90032-3 3661275

[B55] Dolcini MM Adler NE Lee P Bauman KE An assessment of the validity of adolescent self-reported smoking using three biological indicators Nic Tob Res 2003 5 473 483 12959785

[B56] Schofield MJ Lynagh M Mishra G Evaluation of a health promoting schools program to reduce smoking in Australian secondary schools Health Educ Res 2003 18 678 692 10.1093/her/cyf048 14654501

[B57] SAS Institute Inc SAS software version 8.2 for windows 2001 New York: Carry, NC

[B58] Liang KY Zeger SL Longitudinal data analysis using generalized linear models Biometrika 1986 73 13 22 10.1093/biomet/73.1.13

[B59] Oberdorfer A Wiggers J Bowman C Burrows S Math B Cockburn J Considine RJ Monitoring and educational feedback to improve the compliance of tattooists and body piercers with infection control standards: A randomized controlled trial Americn Journal of Infection Control 2004 32 147 154 10.1016/j.ajic.2003.07.005 15153926

[B60] Wolfenden L Wiggers J Knight J Campbell E Spigelman A Kerridge R Moore K Increasing smoking cessation care in a preoperative clinic: a randomized controlled trial Prev Med 2005 41 284 290 10.1016/j.ypmed.2004.11.011 15917023

